# Repeatability of ocular surface vessel density measurements with optical coherence tomography angiography

**DOI:** 10.1186/s12886-019-1255-2

**Published:** 2019-12-10

**Authors:** Sijie Cai, Fengping Zhao, Chixin Du

**Affiliations:** 1 Department of Ophthalmology, First Affiliated Hospital, College of Medicine, Zhejiang University, Zhejiang, Hangzhou China; 2 Department of Ophthalmology, Fourth Affiliated Hospital Zhejiang University School of Medicine, Yiwu, 322000 Zhejiang China; 3 Department of Ophthalmology, Yiwu Traditional Chinese Medicine Hospital, Jinhua, 322000 China

**Keywords:** Ocular surface, Optical coherence tomography angiography, Pinguecula, Pterygium, Vessel density

## Abstract

**Background:**

To determine the repeatability of measurements of ocular surface vessel density in normal and diseased eyes using optical coherence tomography angiography (OCTA).

**Methods:**

Ten normal eyes, 10 pinguecula eyes, and 10 pterygium eyes of 30 volunteers were subjected to OCTA (AngioVue Imaging System, Optovue, Inc.). For scanning, we used the corneal adapter module. Each eye was scanned three times in the nasal and temporal directions, separately. AngioVue software was used to generate the ocular surface vessel density. Ocular surface vessel density was defined as the proportion of vessel area with blood flow to the total measurement area (3 × 3 mm^2^). Intersession repeatability of the measurement was summarized as the coefficient of variation (CV), and intraclass correlation coefficients (ICC) were calculated by variance component models.

**Results:**

The CVs were less than 5% in all subjects, and the ICCs exceeded 0.9; thus, all measurements showed good repeatability. The nasal vessels densities differed significantly between healthy eyes and eyes with pterygium (*P* < 0.05); however, there was no significant difference between healthy eyes and eyes with pinguecula (*P* = 0.466).

**Conclusions:**

These results suggest that measurement of ocular surface vessel density by OCTA in normal eyes and eyes with pterygium and pinguecula is repeatable. This preliminary research describes a quantitative and visual method for assessing vessel density of the ocular surface with a high level of consistency.

## Background

The ocular surface includes the conjunctiva, cornea, and episclera. The conjunctiva is a densely vascularized tissue covering the eye, but the cornea lacks blood vessels [1]. Conjunctival diseases, such as pinguecula and pterygium, are commonly encountered in ophthalmology outpatients. In eastern China, the prevalence of pinguecula has been reported to be 75.57% in people over 50 years of age, which is higher than that reported in previous studies [2]. The prevalence of unilateral cases of pterygium reported from different parts of the world ranges from 0.3 to 37.1% [3]. Many conjunctival and corneal diseases have different degrees of vascular hyperplasia, including pterygium, conjunctivitis, conjunctivoma, keratitis, corneal pannus, and contact lens related inflammation. Judging the extent of neovascularization is necessary in clinical diagnosis. A reliable method for calculating the vessel density could thus play an important role in analyzing and managing these diseases.

Furthermore, red blood cell movement can be directly and non-invasively visualized within the conjunctival vessels, which is not possible in the rest of the human body. Blood flow changes in the bulbar conjunctiva may also represent the vascular flow in other body tissues, such as the cerebral blood flow, as the ophthalmic artery, which supplies the bulbar conjunctiva, originates from the internal carotid artery [4, 5]. It has been reported that the conjunctival vessel changes are similar to well-known vessel changes seen in the retina of patients with diabetes [6]. Therefore, measuring conjunctival vessel changes might provide an indirect method to appraise vascular flow in related body tissues.

Optical coherence tomography angiography (OCTA), a method that uses motion contrast imaging of erythrocyte movement to visualize vessels, has been recognized as a helpful tool for providing a rapid, noninvasive, quantitative evaluation of the retina and choroid or optic nerve vessels. OCTA can further resolve superficial and deep retinal vascular networks, separately. Recent studies have also shown that it is possible to use OCTA with a corneal adapter module (CAM) [7] to image and evaluate the structure of the anterior segment. OCTA can also provide high-resolution imaging and good delineation of the ocular surface vessels by using the tracing algorithm in the OCTA system [8]. Nevertheless, the use of OCTA for measuring ocular surface vessel density has not been investigated to date.

Thus, to assess the usefulness of OCTA in this regard, in this study, we determined the intra-session repeatability of ocular surface density measurements by OCTA in patients with and without pinguecula or pterygium.

## Subjects and methods

Our study followed the principles of the Declaration of Helsinki. Written informed consent was obtained from each patient after explanation of the purpose of the study and before examinations. This study was approved by the institutional review committee of Fourth Affiliated Hospital Zhejiang University School of Medicine; the approval number is K201800023.

To verify the repeatability of ocular surface vessel density measurements with OCTA, we performed OCTA and slit-lamp photography examination in 30 eyes from 30 volunteers at the Fourth Affiliated Hospital Zhejiang University School of Medicine, from January 2018 to March 2018. The average age of volunteers in the normal, pinguecula, and pterygium groups were 49.03 (range: 33–59), 51.00 (range: 33–61), and 53.03 (range: 37–62) years, respectively.

The subjects who underwent OCTA examination had no history of keratitis, conjunctivitis, scleritis, uveitis, or glaucoma, which might cause abnormal vascular hyperplasia. No participants were contact lens wearers. No eyes included in the study received treatment, such as vaso-excitor eye drops, or had undergone any ophthalmological operation or ocular trauma. All subjects underwent slit-lamp photography. Ocular surface vessel density examinations were performed using the AngioVue OCTA system (Optovue, Inc., Fremont, CA) with the CAM and the motion correction technique (MCT) was used to correct motion artifacts [9]. The AngioVue system uses a light source centered at 840 nm and a bandwidth of 50 nm, with an axial resolution of 5 μm and a beam-width of 22 μm [10], and captures two consecutive B-scans containing 304 × 304 A-scans at 70000 scans per second. From a 3–4-s examination, a 3-dimensional scan cube of a 3 × 3 mm^2^ area can be constructed [11]. The split-spectrum amplitude-decorrelation algorithm was used to extract vasculature information.

Volunteers underwent OCTA scanning as follows. The subjects were asked to fixate on the external fixation target with their eyes wide open and were allowed a 1-min break between scans. When scanning the nasal conjunctiva, the fixation target light was set in front of the eyes at the temporal side, and when examining the temporal conjunctiva, the target light was set at the nasal side. Because the system was designed for the retina, we needed to adjust the YXZ focal lengths to ensure that the conjunctiva was clearly seen on the camera image. Then, we set the Z Motor, Focus Value, and P Motor manually to ensure that the scope focused on the conjunctival vessels and to maximize the signal intensity. An independent trained operator scanned the cube regions temporal to the limbus and nasal to the limbus; these areas have a high incidence of pinguecula and pterygium, and it is easy to fix the scope position in these areas, given the color difference between the limbus and conjunctiva.

**Fig. 1:**
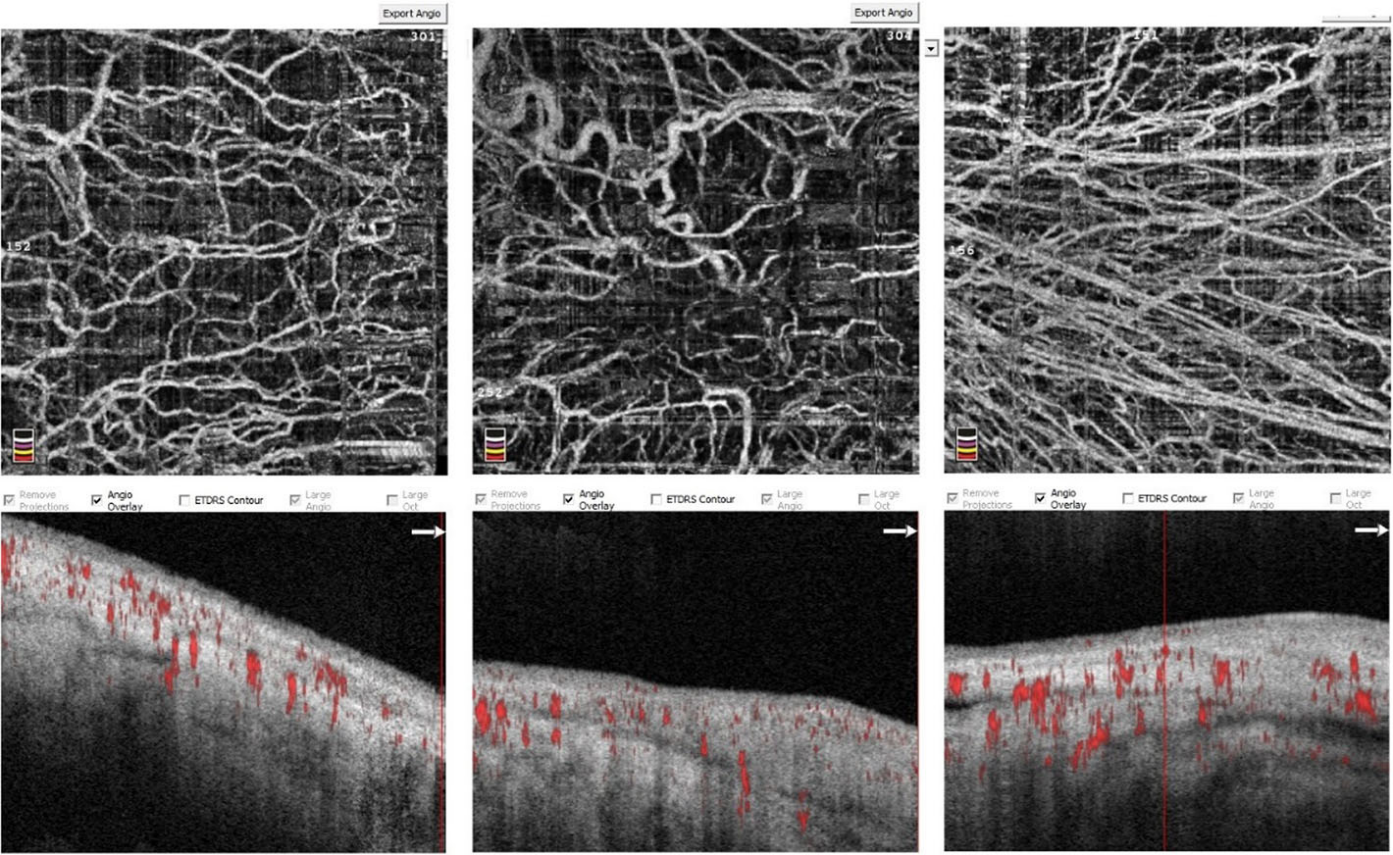
Representative OCTA-based measurement of ocular surface vessel density on a 3 × 3 mm^2^ scan. The left upper panel shows the angiography images of the scanning area in normal eyes. The red dots on the lower panel mark the tomographic vessel signals. The middle two panels represent an eye with pinguecula, and the right two panels represent an eye with pterygium

Each side was scanned three times to assess the intrasession repeatability. The image quality score (0–10) was calculated by the built-in software (Re Vue, version 2014.2.0.65; Optovue, Inc.) based on the following criteria: Signal Strength Index (SSI) [12] and artifacts due to eye motion or eyelid margins, and the coincidence rate of two orthogonal scans. The SSI ranges from 1 (poor) to 100 (good); high quality images were defined by an image score of at least 6. Ocular surface vessel density was defined as the proportion of vessel area with blood flow to the total measurement area (3 × 3 mm^2^) (Fig. 1).

### Statistical analysis

Statistical analyses were conducted using SPSS version 22 (SPSS, Inc., Chicago, IL) statistical software. Descriptive statistics included the mean value, standard deviation (SD), range, percentages, coefficients of variation (CVs), intraclass correlation coefficients (ICCs) and 95% limits of agreement (LoAs). Bland and Altman and the British Standard Institution [13] have proposed the use of the CV and ICC to express the repeatability of different properties. CV was calculated as the pooled between-measurement SD divided by the mean of the measurements and was expressed as a percentage [14]; small CVs (< 5%) indicate good repeatability. ICCs (ranging from 0 to 1) were calculated by estimating the proportion of the total SD of a measurement explained by the actual measurement differences [12]. Large ICCs (> 0.9) indicate smaller fluctuations between repeated measurements within an individual eye than between eyes. A 95% confidence interval was utilized, and results with a *P* value ≤0.05 were considered significant.

## Results

The CVs and ICCs for each group and the mean values of vessel density of the study participants are presented in Table 1. Thirty eyes of 30 volunteers were enrolled in the study, including 10 normal eyes, 10 eyes with pinguecula in the nasal region, and 10 eyes with pterygium in the nasal region. The mean SSI for the different groups varied from 76.65 to 86.64, and the image quality score ranged from 7.61 to 8.59, indicating good image quality.

**Table 1:** Repeatability of measurements of ocular surface vessel density

	Image quality score (mean ± SD)	Signal strength index (mean ± SD)	ICCs	0.95% Confidence interval of ICCs	CV (%)	Vessel density (mean ± SD)
Normal eyes (nasal)	8.24 ± 1.61	81.58 ± 13.55	0.902	0.712–0.973	3.72 ± 0.93	51.88 ± 4.10
Normal eyes (temporal)	7.91 ± 1.62	85.15 ± 16.49	0.976	0.930–0.994	3.31 ± 0.56	51.13 ± 6.67
pinguecula eyes (nasal)	7.61 ± 1.61	83.74 ± 10.36	0.972	0.919–0.993	2.58 ± 0.57	50.35 ± 4.81
pinguecula eyes (temporal)	8.44 ± 1.61	86.64 ± 11.24	0.970	0.913–0.992	3.72 ± 0.55	49.40 ± 6.41
pterygium eyes (nasal)	8.59 ± 1.12	76.65 ± 12.83	0.931	0.798–0.981	2.41 ± 0.49	64.08 ± 3.78
pterygium eyes (temporal)	7.63 ± 1.67	83.57 ± 15.60	0.978	0.937–0.994	3.07 ± 0.36	52.22 ± 5.75

The intrasession ICCs for all groups exceeded 0.9 (range: 0.902–0.978), showing excellent repeatability for the measurement of ocular surface vessel density. The group of eyes that had normal nasal regions had the lowest ICC value, and the group with pterygium in the temporal region had the maximum ICC value (0.902 and 0.978, respectively). In addition, the ICC in the temporal region of normal eyes was 0.976, which was higher than the corresponding value of 0.902 in the nasal region of normal eyes. Similarly, the ICCs tended to be substantially higher for measurements of temporal vessel density than for nasal regions in pterygium eyes.

CVs indicate repeatability by calculating the variability of several measurements for the same subject. In general, the CVs were < 5%, showing good repeatability. The CV of the group with normal nasal regions and pinguecula temporal regions (3.72 ± 0.93% and 3.72 ± 0.55%, respectively) was higher than that of other groups, while the CV was lowest in the group with nasal pterygium (2.41 ± 0.49%). Furthermore, the mean density of eyes with nasal pterygium was significantly higher than that of other groups (64.08 ± 3.78, *P* < 0.05).

## Discussion

Currently, slit-lamp photography or angiography techniques employing fluorescein or indocyanine green [15] are the primary methods of evaluating the conjunctival and corneal vasculature. However, these angiography techniques are invasive and non-quantitative. In terms of clinical application, OCTA represents an accurate, quantitative, and noninvasive method for diagnosing fundus diseases and monitoring vascular changes before and after treatment. The current OCTA system uses the split-spectrum amplitude-decorrelation angiography concepts (SSADA) [16] to collect data about the vasculature. The SSADA method is used when performing B-scans repeatedly in the same area, to catch motion contrast and thereby make vasculature visible (by catching signal changes resulting from flowing erythrocytes). Using the MCT, artifacts that remain in the OCTA can be eliminated [9]. The artifacts may result in worse repeatably of measurements.

Use of OCTA for evaluation of anterior segment vasculature and even corneal vessels has been reported [7]. However, OCTA has not been prospectively applied to monitor the ocular surface, to date. The clinical utility of an instrument is highly dependent on the repeatability of its measurements. Repeatability refers to the closeness of the agreement between the results of multiple tests for single volunteers under the same conditions. Assessment of a method’s repeatability is essential for determining its reliability for application in clinical management and for research. The current study showed good intrasession repeatability for OCTA-based measurements of ocular surface vessel density in all the groups.

Pterygium is a disease commonly encountered in the clinic [15]; it is a thickened triangular layer of conjunctiva extending from the nasal or temporal edge of the eye to the cornea. Pterygium typically requires surgery when its growth becomes unmanageable or causes visual problems [17]. Pinguecula is histologically similar to pterygium [18]. It can be difficult to distinguish manageable pterygium and unmanageable pterygium by using slit-lamp photography. In pinguecula, increases in the amount of constituent substances, such as degenerating collagen fibers, variant ground substance, abnormal conjunctival cells, and areas of increased electron density, are major sources of pinguecula growth [19]. There is no vascular hyperplasia in pinguecula, while the pterygium has been confirmed to have a high degree of vascularity [20]. OCTA enables high-resolution qualitative and quantitative imaging of pterygium and could allow observation of changes over time, which could enhance the accuracy of diagnosis and facilitate determination of the appropriate treatment regimen.

In previous studies, the pterygium was shown to arise from irritation of the pinguecula. In clinical practice, pterygium and pinguecula are sometimes difficult to distinguish. However, the difference between the conjunctival vessel density, as determined by OCTA, of the pterygium and pinguecula was marked (*P* < 0.05); there was no significant contrast between normal eyes and pinguecula eyes in terms of the density of the nasal ocular surface vessels (*P* = 0.466). This might be because the vascular proliferation in pterygium and the hyaline degeneration [20] has no impact on vessel density measurement.

In our study, the nasal pterygium group had both the lowest CVs and the maximal vessel density. In contrast, the group with pinguecula temporal regions had maximal CVs and the lowest vessel density. This suggests that vessel density and CVs are positively correlated; this may be related to how the SSADA algorithm distinguishes vessels, by visualizing the ocular surface blood flow. Specifically, the blood flow results in fluctuations in the amplitude of OCT fringes (speckle) as red blood cells (RBCs) move within a particular voxel [21]. The bigger vessel density allows more RBCs in the particular voxel, which might leads to great measurement stabilization and repeatability. However this conclusion requires further data for verification. We use the method described by Carkeet [22] to calculate the exact confidence intervals for Bland-Altman analysis for intrasession repeatability of vessel density measurement made on the temporal pinguecula group and the nasal pterygium group. The mean of differences was 1.48% (with SD_diff_ of 5.62%) for the temporal pinguecula group; the LoAs are shown to be 12.51% (confidence interval, 9.31 to 22.70%) and − 9.45% (confidence interval, − 6.34% to − 19.72%). The mean of differences was 0.17% (with SD_diff_ of 3.76%) for the nasal pterygium group; the LoAs are shown to be 7.54% (confidence interval, 5.40 to 14.35%) and − 7.20% (confidence interval, − 5.06% to − 14.00%). According to the analysis above, it is difficult to claim that 95% LoAs are lower in the nasal pterygium group due to the overlap between the confidence intervals.

Many factors can decrease measurement stability and thus repeatability, such as movement of the eyes or unstable fixation. However, this effect could be minimized by rapid and consistent positioning. A vertical flare can also cause a strong reflection on the vertex of the conjunctiva, and the resulting slight signal peaks might cause measurement errors. In addition, the eyelid might generate pressure on the ocular surface vessels, which could influence RBC movement when blinking.

The study has some limitations. A new measurement method needs to be validated; however, this method has not been compared to other methods used to measure ocular surface vessel density, and the study lacks a reliable gold standard for measurements. This issue should be addressed in future studies. Second, the types of disease and age range of the subjects were relatively limited and should be expanded. Some types of keratoconus are associated with corneal neovascularization, and the repeatability of corneal vessel measurements should be investigated in future studies. Third, OCTA can measure the retinal vessels layer by layer. In the future, the superficial vascular network and the deep layer vascular network could be assessed separately. Three-dimensional technology could also be added to the method to reconstruct stereoscopic blood vessels. Moreover, the scanning scope used in the method is 3 × 3 mm^2^, and repeatability may be affected by expansion of the scope.

## Conclusions

OCTA is a rapid, in vivo, noninvasive, and quantitative imaging system that can be used not only for assessing retinal vasculature but also for ocular surface vascular measurements in healthy eyes or eyes with conjunctival disease. This study demonstrated that the technology has a high level of repeatability and can be used in clinical diagnosis and pathophysiological investigations of ocular surface diseases. Further research is needed to increase the speed and sensitivity of the method, verify its validity, and evaluate its ability to measure the ocular surface vessel density in different layers.

## Data Availability

The datasets used and/or analyzed during the current study are available from the corresponding author on reasonable request.

## Abbreviations

CV: coefficient of variation
ICC: intraclass correlation coefficients
LoAs: 95% limits of agreement
MCT: motion correction technique
OCTA: optical coherence tomography angiography
RBC: red blood cells
SD_diff_: SD of the differences
SSADA: split-spectrum amplitude-decorrelation angiography concepts
